# A thermostable DNA primase‐polymerase from a mobile genetic element involved in defence against environmental DNA

**DOI:** 10.1111/1462-2920.15207

**Published:** 2020-09-03

**Authors:** Nieves García‐Quintans, Ignacio Baquedano, Alba Blesa, Carlos Verdú, José Berenguer, Mario Mencía

**Affiliations:** ^1^ Centro de Biología Molecular Severo Ochoa (CBMSO) Universidad Autónoma de Madrid‐Consejo Superior de Investigaciones Científicas Madrid 28049 Spain; ^2^ Department of Biotechnology, Faculty of Experimental Sciences Universidad Francisco de Vitoria Madrid 28223 Spain

## Abstract

Primase‐polymerases (Ppol) are one of the few enzymes able to start DNA synthesis on ssDNA templates. The role of *Thermus thermophilus* HB27 Ppol, encoded along a putative helicase (Hel) within a mobile genetic element (ICETh2), has been studied. A mutant lacking Ppol showed no effects on the replication of the element. Also, no apparent differences in the sensitivity to DNA damaging agents and other stressors or morphological changes in the mutant cells were detected. However, the mutants lacking Ppol showed an increase in two to three orders of magnitude in their transformation efficiency with plasmids and genomic DNA acquired from the environment (eDNA), independently of its origin and G + C content. In contrast, no significant differences with the wild type were detected when the cells received the DNA from other *T. thermophilus* partners in conjugation‐like mating experiments. The similarities of this behaviour with that shown by mutants lacking the Argonaute (ThAgo) protein suggests a putative partnership Ppol‐ThAgo in the DNA–DNA interference mechanism of defence, although other eDNA defence mechanisms independent of ThAgo cannot be discarded.

## Introduction

Primase polymerases (Ppol thereafter) are enzymes belonging to the Archaeo‐Eukaryal primases familiy (AEPs). They are among the few enzymes that synthesize DNA *de novo* using single stranded DNA as template (Guilliam *et al*., 2015). These enzymes were first discovered in Archaea, but it was later found that they form a much larger group with great internal diversity distributed among the three domains of life and also present in viruses (Kazlauskas *et al*., 2018). For the few representatives already studied, a panoply of different functions has been ascribed, from replication in phages to diverse mechanisms of DNA repair, like the re‐start of stalled replication forks or translesion synthesis by polymerizing on single‐stranded DNA templates (Boldinova *et al*., 2017).

Ppol‐like genes of prokaryotes are most frequently located within mobile genetic elements (Kazlauskas *et al*., 2018). The thermophilic Ppol protein from *T. thermophilus* HB27 (Tth HB27) has been characterized in great detail (Picher *et al*., 2016) for its commercial use in combination with the Φ29 DNA polymerase in kits for whole‐genome isothermal amplification (TruePol amplification, 4BasesBio). We have shown recently that the gene encoding this protein belongs to ICETh2, a mobile genetic element integrated into the chromosome of Tth HB27 (positions 641 829–653 145) at a Val‐tRNA gene (Baquedano *et al*., 2020). ICETh2 can excise at low frequency from the chromosome (10^−4^ to 10^−5^) under normal growth conditions and integrate at a copy of its attachment site supplied *in trans* in a plasmid. Both excision and integration depend on the activity of a recombination‐specific tyrosine recombinase (Int2), which is also responsible for the excision and integration of ICETh1, the first mobile genetic element described in Tth HB27, integrated in this case at an Ile‐tRNA gene (positions 1 778 501–1 793 358). ICETh1 contains genes that are critical for the DNA donation capability of the host strain to naturally competent partners during *transjugation*, the unconventional type of conjugation described for Tth (Blesa *et al*., 2017). Since excision increases their transfer frequency, both ICEs became interdependent for their successful transfer to other cells (Baquedano *et al*., 2020). This interdependence, along with the absence of putative replication‐related enzymes coded in ICETh1, lead us to suggest for *ppol* and maybe for its surrounding genes, a putative role in replication and/or spread of these mobile elements, as suggested for proteins of the Ppol family identified within putative mobile genetic elements (MGE) from Bacteria and Archaea (Kazlauskas *et al*., 2018).

In this work, we analyse the role of Ppol in the biology of ICETh2 and of its host, Tth HB27. We show that mutants lacking Ppol have no detectable defects at the phenotypic level, except for a sharp increase (>10^2^‐fold) in the transformation efficiency of the strain by natural competence, being this increase higher for integrative plasmids than that observed for mutants lacking the argonaute (ThAgo) protein. As it happens with the ThAgo protein (Blesa *et al*., 2015), this barrier function is not active when the DNA is transferred through the DNA donation machinery encoded by ICETh1. A putative collaboration between Ppol and ThAgo in DNA interference is also suggested.

## Results

### Distribution of ICETh2 homologues

**Fig 1:**
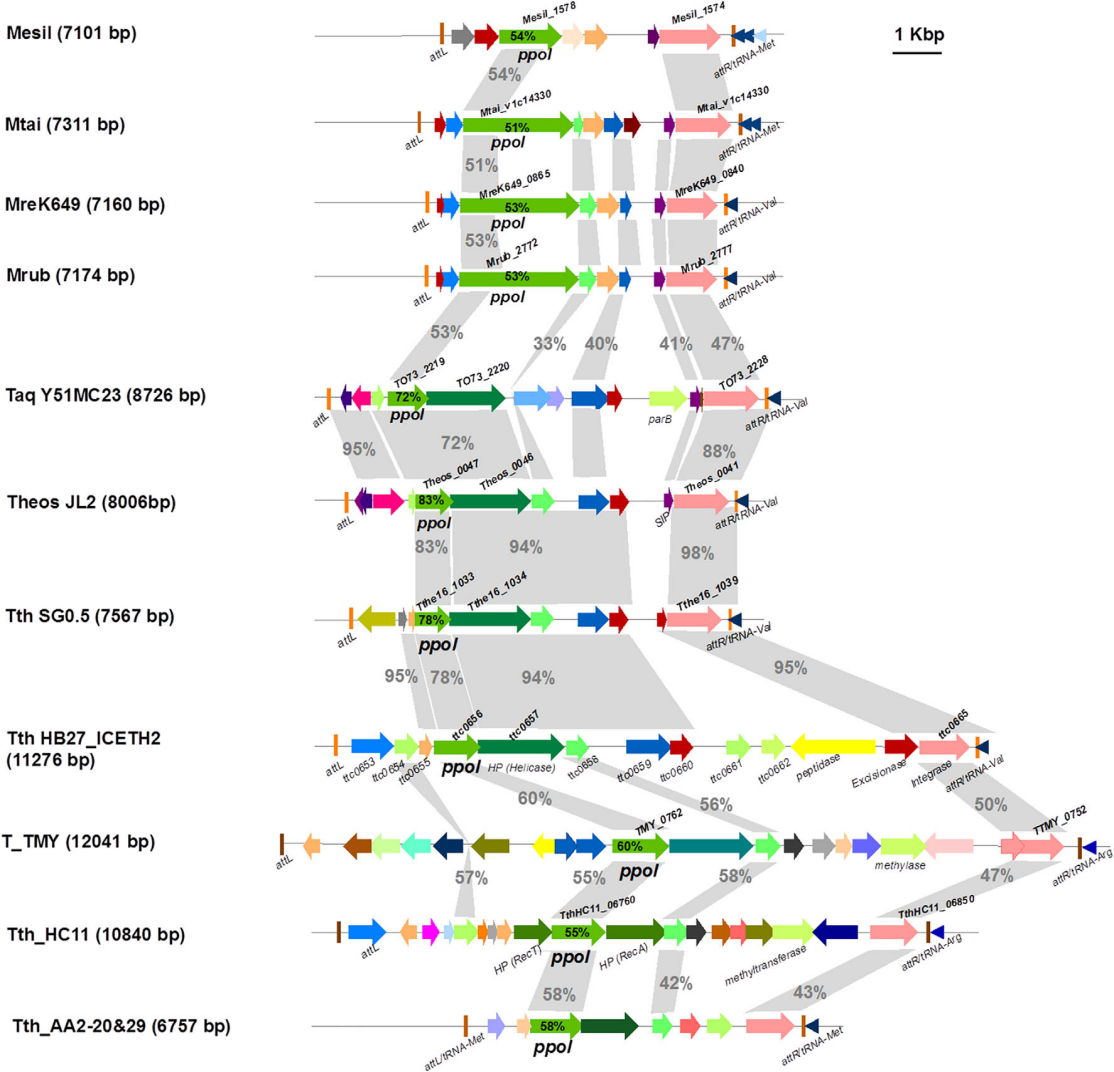
Genetic context of *ppol* within MGE elements of *Thermales*. Selected genome maps of homologues to the ICETh2 structure defined in *T. thermophilus* HB27 strain in (Baquedano *et al*., 2020), employing available data in the KEGG and NCBI databases. ICETh2 is an MGE mobilized by a set of highly conserved integrase (in pale pink) and an excisionase (in red) followed by a tRNA‐Val at the 3′ termini. This 11.3 Kbp ICETh2 harbours a putative replication module containing several hypothetical proteins, Ppol (in green) and a putative helicase gene (in dark green); a regulation module including a peptidase (in yellow), putative transposases (in blue navy) and other ORFs coding for hypothetical proteins including helicase‐like motifs (in light green) which may contribute to the physiology of this MGE. Flanking this ICE element there is a 47 nt direct repeat (*att* sites, in orange) identical to the 3′ end of the tRNA, which show high homology among all strains. A type II toxin (purple)‐ antitoxin (pink) (HicA‐B type) is detected in certain strains. Loci of the Ppol and the helicase ‐like genes as well as the integrase are annotated in each of the ICEs identified. Sequence identity percentages to ICEth2 genes are given in the corresponding arrows. Highly similar structures to that of ICETh2 are found in *T.aquaticus* (Taq), *T.oshimai* (Theos) and *T. thermophilus SG0.5 JP17‐16* (SG0.5) and with a lower synteny in *T. thermophilus* TMY (T_TMY), HC11 (Tth_HC11) and AA2 strains, the later showing two identical ICEs (AA2‐20 and AA2‐29). In these strains, the helicase is replaced by an hypothetical protein containing a P‐loop‐NTPase on its N‐termini (in darker green). Besides, several strains belonging to the *Meiohermus* genus: *M. silvanus* (Mesil), *M. taiwanensis* (Mtai) and two strains of *M. ruber*, DSM1279 (Mrub) and K649 (Mre) also harbour Ppol homologues within an ICE‐like structure. Search was done on fully assembled genomes available on NCBI databases, selecting those that fulfilled the identity and coverage established (40% identity, 60% coverage, within a 30 kbp distance).

ICETh2 is 11 276 bp long and encodes 13 ORFs, most of them corresponding to hypothetical proteins with little homology to proteins of known function. However, there are mutant‐based *in vivo* studies on Int2 and the associated excisionase (Exc2) proteins that show their requirement for excision and intracellular mobilization of ICTEh2 and ICETh1 (Baquedano *et al*., 2020). Two other ORFs encoded downstream of *ppol* have homologues in the Genbank, one to proteins with putative DNA helicase activity (TTC_0657, named Hel thereafter) and another one annotated as putative peptidase (TTC_0663) (Fig. 1).

A search for putative MGE related to ICETh2 was carried out by looking in GenBank and KEGG databases in fully assembled genomes for the presence of protein homologues to Ppol, Hel, and Int2, with at least 40% of amino acid sequence identity and 60% coverage that were located within 30 kb distance between them. In particular, the distance between Ppol and Hel coding genes was set to a maximum of 10 kb, whereas a longer distance (20 kb) was allowed for homologues of Int2 due to their frequent location near one of the extremes of the mobile element. Further search for the presence of tRNA genes in the vicinity of the Int2‐homologue and a 45–50 bp repeat identical to the 3′ extreme of the tRNA at the other extreme of the DNA encoding these three gene homologues was also used to identify the sequence as a mobile element.

This search allowed for the identification of putative mobile elements related to ICETh2 in the genomes of *T. aquaticus* Y51MC23 (8726 bp ICE length), *T. oshimai* JL2 (8006 bp), and *T. thermophilus* SG0.5JP17‐16 (7567 bp), and with a lesser sinteny and homology in *Thermus* TMY (12,041 bp), and in the *T. thermophilus* strains HC11, AA2‐20 and AA2‐29 (Fig. 1). In four *Meiothermus* spp., the homology to Ppol was limited to the N‐terminal domain of much larger proteins (970–1000 amino acids long), with a C‐terminal region containing a DUF927 domain. A DUF927 domain from *Staphylococcus aureus* has been shown to have helicase activity (Mir‐Sanchis *et al*., 2016). Besides, other putative ICEs lacking Ppol such as the one found in Tth HB8 and MGE‐like clusters with no clear borders, sharing homologues to Ppol, Hel and Int2, were found also in other *Thermus* strains (Supporting Information Fig. S1) and other thermophilic genera (Supporting Information Table S1).

The identity between the Ppol from ICETh2 and that coded by the putative ICEs identified in our analysis followed a phylogenetic relationship, ranging from 50% of identity for *Meiothermus* homologues to the 83% identity shared by *T. oshimai*. The gene immediately downstream of *ppol* in ICETh2 (*hel*) was also highly conserved in the putative mobile elements identified, ranging from 72% to 95% of sequence identity to the HB27 corresponding proteins. Nonetheless, the highest identity was found for the integrases, with 88%–97% identity to Int2 within the *Thermus* genus, and lower identities (47%) in those found in *Meiothermus* spp.

In addition, the ICETh2‐related MGE identified encoded a number of putative ORFs of unknown function also conserved in the other ICE‐like structures, like homologues of HB27 TTC0655 found in Tth SG0.5, and homologues to TTC0658, TTC0659 and TTC0660 encoded by most of the putative ICEs identified.

The conserved presence in these putatively mobile elements of a Ppol homologue followed by a gene encoding a putative helicase or a single protein encompassing both domains suggested roles for these proteins as part of a hypothetical replication module, as proposed for homologues of these proteins found in putative MGE of Bacteria and Archaea (Kazlauskas *et al*., 2018).

### Ppol is not required for ICETh2 replication

**Fig 2:**
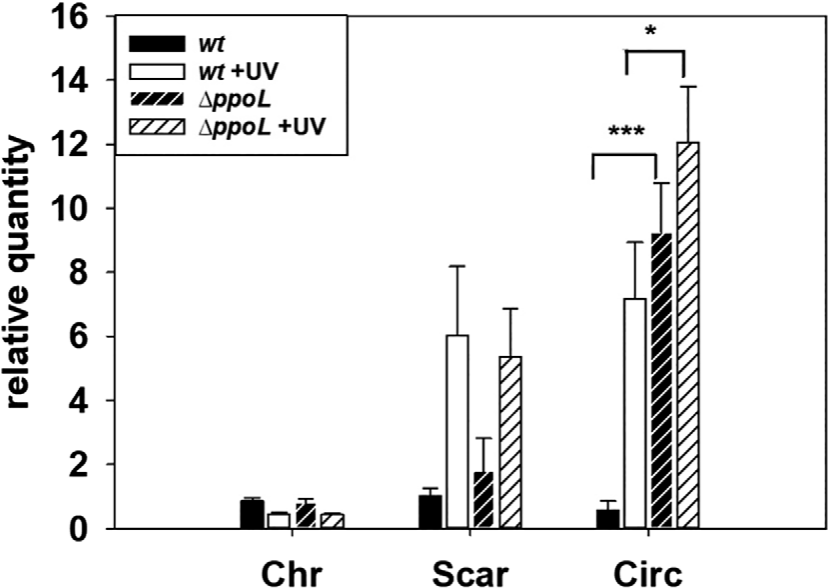
An insertional deletion of *ppol* increases the excision of ICETh2. Relative quantities detected by qPCR of the integrated ICETh2 *attL2* form (Chr), the circular excised *attI2* form (Circ), and the ICE‐free genomic locus *attB2* (Scar). The ratios are shown for the wild type and the Δ*ppol::kat* mutant subjected to UV treatment respect to untreated controls. First replicate of wild‐type cultures have been normalized to 1 on each form. Asterisks indicate significant statistical differences (*P‐value < 0.05 and ***P‐value < 0.01) between wild type and mutant ratios under the same conditions for the detection of the same ICE form. Samples have been normalized relative to the RNA polymerase alpha subunit gene (*n* = 5).

Under normal growth conditions, ICETh2 excises in stationary phase at very low frequency (aprox. 4 × 10^−5^), and the copy number of the excised form is similar to that of the ICETh2‐free genomic locus (Scar) (Baquedano *et al*., 2020). However, and having in mind that excision and replication of ICEs is frequently induced by DNA stress (Johnson and Grossman, 2015), we checked for the possibility of ICETh2 replication under UV‐generated DNA damage, and the putative role of Ppol in this process, as well as other Ppol roles in the context of Tth HB27 biology. To this end, we isolated a Δ*ppol::kat* mutant through homologous recombination of the chromosomal gene with a construct in which the *ppol* gene was replaced by a cassette (*kat*) conferring thermostable resistance to Kanamycin (Kn^R^) and submitted the wild type and this derivative mutant to UV treatment (Experimental procedures section). Then, total DNA from both strains was extracted, and the relative amounts of chromosome‐integrated (Chr), circular excised forms (Circ), and of the ICETh2‐free chromosome (Scar), were determined by qPCR in comparison with untreated cultures (Fig. 2).

The data show that, in the wild type, the Circ and the Scar forms are induced fivefold to sevenfold by UV treatment. Concomitantly, the ratio of the inserted form also appears to decrease with treatment, but the differences were not significant enough. Notwithstanding, these results show that the excision of ICETh2 is increased under UV‐stress, as it happens in other ICEs, but this excision is not followed by a significant replication of the element, as the increase detected in the Scar and in the Circ forms are apparently similar.

Interestingly, in the *ppol::kat* mutant, in the absence of UV treatment, the Circ form was eightfold to 10‐fold higher than in the wild type. In contrast, the level of the Scar was only twice that of the wild type. This suggests that replication of ICETh2 could take place in the absence of Ppol. Under UV treatment, the amount of Scar copies increased by twofold to threefold in the *ppol* mutant, approximately similar to the wild type case, but the treatment did not significantly increase the amount of the Circ form, in contrast to what happens in the wild type. In conclusion, (i) replication of ICETh2 after excision is quite low if existent at all under normal conditions, or even under UV treatment; (ii) excision of the element without concomitant replication is clearly stimulated by UV in the wild type; (iii) *ppol* insertional deletion produces a sharp increase in the excised form and this increase is not significantly altered by UV treatment, and (iv) replication of the excised ICETh2 can be suggested to happen in the Δ*ppol* strain in the absence of UV.

### Ppol deletion has no major phenotype under regular or stressed conditions

**Fig 3:**
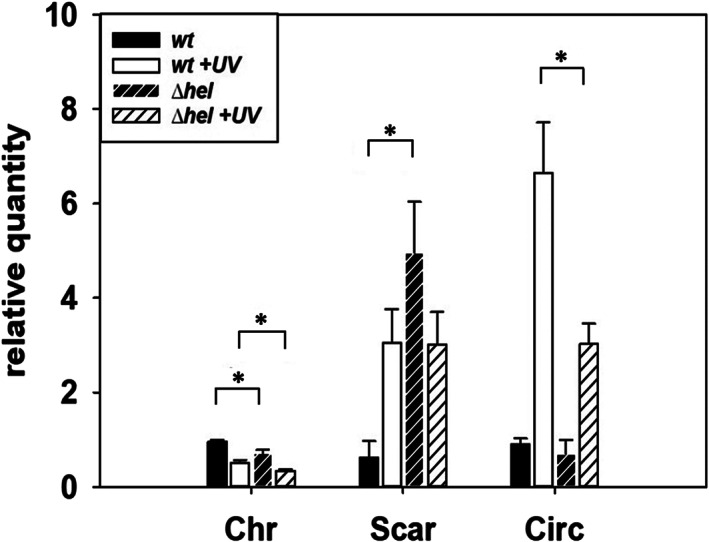
An insertional deletion of Hel increases the proportion of ICE‐free form. Relative quantities detected by qPCR of Chr, Circ, and Scar forms. First replicate of wild type cultures has been normalized to 1 on each form. Asterisks indicate significant statistical differences (*P*‐value < 0.05) between wild type and mutant ratios under the same conditions for the detection of the same ICE form. Samples have been normalized relative to RNA polymerase alpha subunit, DNA Polymerase III and 16S genes (*n* = 3).

The results above support that Ppol has no significant role in ICETh2 replication. At the same time, the results suggest that Ppol absence mirrors a stress condition of DNA damage. This could be related to putative polar effects of the *kat* gene, with no transcription terminator, on the downstream genes, mainly the putative helicase (Hel) encoded immediately downstream of *ppol*. To analyse this, we isolated a Δ*hel::hyg* mutant and assayed by qPCR the amount of the different ICETh2 forms. The results (Fig. 3) show that the mutant lacking the Hel protein produces levels of the Circ form similar to those in the wild type under control conditions, but a much limited induction of this form after UV treatment. Therefore, the increased excision of the ICETh2 in the *ppol::kat* mutant under unstressed conditions shown in Fig. 2 is likely dependent on the Hel protein, supporting also that its overexpression by polar effects could be the origin of the stressed‐like behaviour detected in Fig. 2 in the Δ*ppol::kat* mutant. On the other hand, the increased amount of the Scar form in the Δ*hel::hyg* mutant (fivefold, Fig. 3) suggests that spontaneous excision could not be compensated by a Hel‐dependent replication and re‐integration of the element in the free *attB* site. Finally, as the Δ*hel::hyg* mutant likely also produces polar effects these data support that the downstream gene (TTC_0663) was not responsible for the apparently stressed phenotype produced in the Δ*ppol::kat* mutant.

**Fig 4:**
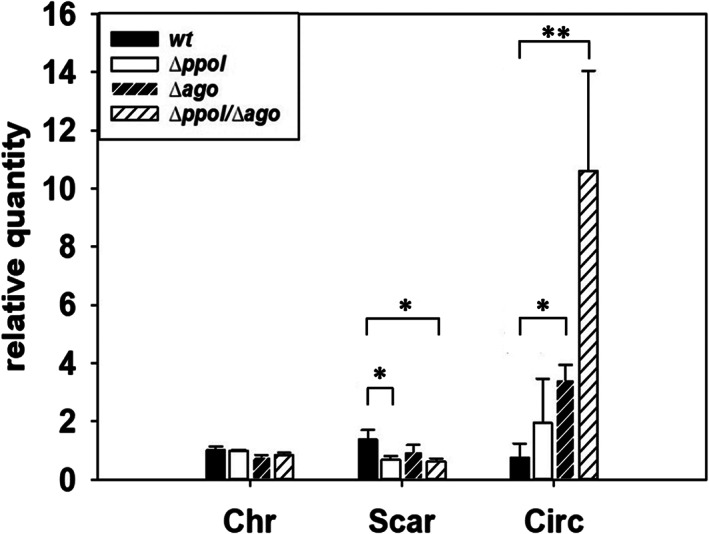
Absence of both Ppol and ThAgo sharply increases ICETh2 excision. Relative quantities detected by qPCR of Chr, Circ, and Scar forms. First replicate of wild‐type cultures has been normalized to 1 on each form. Asterisks indicate significant statistical differences (*P‐value < 0.05 and **P‐value < 0.01) between wild type and mutant ratios for the detection of the same ICE form. Samples have been normalized relative to RNA polymerase alpha subunit, DNA Polymerase III and 16S genes (*n* = 3).

To avoid further interferences produced by polar effects of the Δ*ppol::kat* mutation, we isolated an in‐frame marker‐free Δ*ppol* derivative (Supporting Information Fig. S2) by using the Cre‐lox strategy (Lambert *et al*., 2007; Togawa *et al*., 2018). In agreement with the overexpression of Hel as the likely origin of the increased amounts of the Circ form in the Δ*ppol::kat* mutant, the qPCR assays of the ICETh2 forms in the Δ*ppol* mutant revealed a limited increase (twofold) of the Circ form respect to the wild type under non‐stressed conditions (Fig. 4). This increase is far from eightfold to 10‐fold increase in this form detected in the Δ*ppol::kat* insertion mutant and could be the consequence of a moderate increase in the expression of Hel, or, on a putative interference of Ppol in the replication of ICETh2. In mutants defective in the Argonaute protein (Δ*ago*), which has a strong effect in decreasing the copy number of replicative plasmids (Swarts *et al*., 2014), a fourfold increase in the amount of the Circ form was detected, supporting a role of this protein in limiting replication of ICETh2. Interestingly, in a double Δ*ago‐*Δ*ppol* mutant, the amount of replicative Circ form of ICETh2 increased by around 10 folds, suggesting the existence of a synergistic effect of both proteins in limiting the replication of ICETh2 in the cells.

On the other hand, the growth of the Δ*ppol* mutant at 60°C, 65°C or 70°C did not show any significant differences respect to the wild type or the Δ*ago* and double Δ*ago‐*Δ*ppol* mutants, as observed from their duplication times (Supporting Information Fig. S3) or growth curves (Supporting Information Fig. S4). Optical and transmission electron microscopy also revealed no major differences in the morphology or size between the wild type and the *ppol* mutants (not shown).

As eukaryotic homologues of Ppol have been implicated in DNA damage repair, we tested the effects of UV radiation (Experimental procedures section) after 5, 10, and 15 s of treatment, with similar results for all the strains (Supporting Information Fig. S5A). We also analysed the effects of chemical agents with different DNA damaging effects (H_2_O_2_, Mitomycin C, Ciprofloxacin, Novobiocin and Bleomycin) without finding any significant difference between the wild type and the Δ*ppol* mutant or its Δ*ago* derivative (Supporting Information Fig. S5B and C).

In conclusion, across all these properties, the behaviour of the Δ*ppol* mutant was basically indistinguishable from that of the parental strain, at least under our experimental conditions, suggesting that Ppol has no major role in the regular cell physiology of this bacterium. These results go in line with the fact that the *ppol* gene is absent from the genomes of closely related strains of *T. thermophilus* such as HB8 (Supporting Information Table S1). However, these data also support the existence of some kind of collaboration between Ppol and ThAgo in keeping under control the replication of the ICETh2.

### Ppol deletion sharply increases natural competence

**Fig 5:**
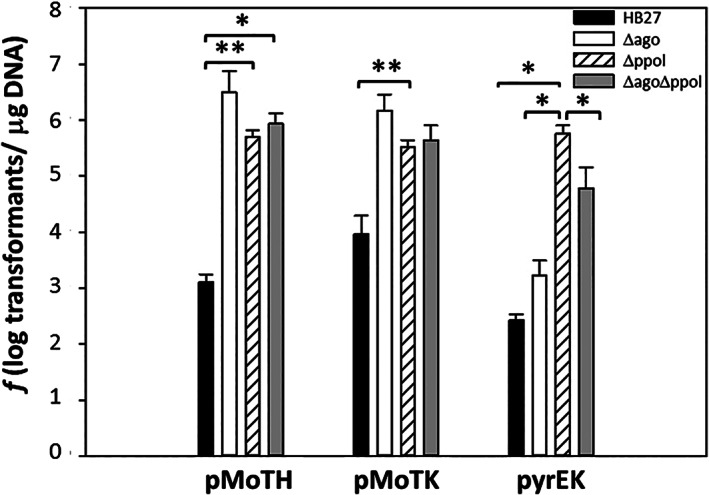
Transformation efficiency of *Ppol* mutants. The 100 ng of replicative plasmids conferring Kn^R^ (pMoTK) or Hyg^R^ (pMoTH), or the suicide plasmid pyrEK (pyrEK) conferring Kn^R^, were used to transform 1.5 × 10^8^ cells of *T. thermophilus* HB27 and its derivative mutants lacking TthAgo (Δ*ago*), Ppol (Δ*ppol*) or both (Δ*ago*‐Δ*ppol*). Transformation is represented as the number of colonies on selection plates at 60°C in three independent experiments. Asterisks indicate significant statistical differences (*P‐value < 0.05 and **P‐value < 0.002).

Having in mind that many ICEs encode different protective functions for their hosts (Rankin *et al*., 2011), we carried out a series of transformation experiments with the wild type strain and the Δ*ppol* mutant. As mutants lacking the ThAgo show an increased transformability (Swarts *et al*., 2014), and the data shown in Fig. 3 suggest some of relationships with Ppol, we also included the single Δ*ago* and the double Δ*ago*‐Δ*ppol* mutants in these assays. Transformation experiments were performed with replicative plasmids pMoTH and pMoTK, conferring thermostable resistances to hygromycin (Hyg^R^) and kanamycin y (Kn^R^), respectively, and an integrative suicide vector to perform double recombination substitution of the *pyrE* gene with the Kn^R^ marker (pyrEK). As shown in Fig. 5, the results revealed a sharp increase in transformability of the Δ*ppol* mutant, as compared with the wild type (compare black bars to stripped ones). This increase reached more than 100‐fold for pMoTK and more than 1000‐fold for pMoTH and for the integrative construct pyrEK. Interestingly, this sharp increase in transformability with the replicative plasmids was similar to that shown by the Δ*ago* mutant, and the absence of both proteins had no additive effects on transformability. However, the Δ*ppol* mutant showed much higher efficiency (100‐fold) in the transformation and recombinative integration of the pyrEK construct respect to the Δ*ago* mutant, which is, in turn, 10‐fold more efficient than the wild type. The Δ*ago*‐Δ*ppol* double mutant showed an increase in transformation and integration of pyrEK intermediate between both single mutants.

In conclusion, our results support that Ppol plays a relevant role in defence against invading DNA. This role is quantitatively similar to that produced by the deletion of ThAgo protein upon transformation with replicating plasmids, but an additional impact for Ppol regarding the integration of suicide plasmids by homologous recombination is also deduced from these data.

**Fig 6:**
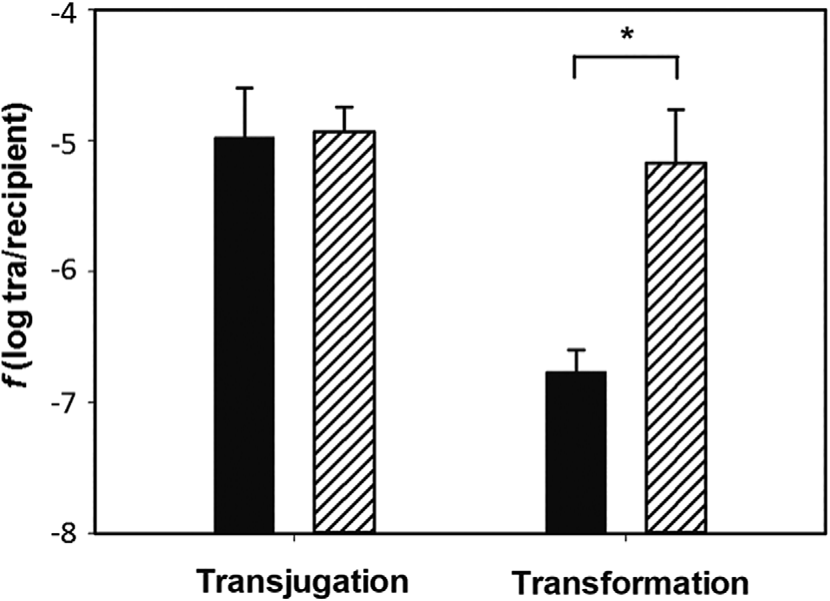
Ppol functions as a barrier against isogenic chromosomal DNA in transformation, but not in transjugation. In transjugation, two wild type strains (black bars), one labelled in the genome at the *pyrE* locus with Kn^R^ and the other labelled at locus TTC0313 (coding for putative ferredoxin‐nitrite reductase) with Hyg^R^ marker, as well as two ∆*ppol* derivatives labelled identically with Kn^R^ and Hyg^R^ (lined bar) were used in mating experiments. For transformation experiments, a wild type strain labelled in the genome at the *pyrE* locus with a Kn^R^ marker (black bar) and a *∆ppol* mutant identically labelled (lined bar) were transformed with 20 ng of genomic DNA isolated from *∆ppol* mutant labelled with a Hyg^R^ marker at the TTC0313 locus. Transjugation and transformation frequencies were calculated as the ratio between the number of double resistant colonies (Kn^R^ plus Hyg^R^) and Kn^R^ colonies. Frequencies are the average of four independent experiments, and error bars correspond to standard deviation. The asterisk indicates significant statistical differences (*P*‐value < 0.05).

In order to check whether Ppol was also active as barrier against isogenic DNA, the wild type and the Δ*ppol* mutant were transformed with chromosomal DNA isolated from Kn^R^ *pyrE::kat* insertion mutants (Table 1). The results of these experiments (Fig. 6, ‘transformation’) showed a 100‐fold increase in transformability of the mutant lacking Ppol with chromosomal DNA, similar to that observed with plasmids conferring Kn^R^ in Fig. 5, thus supporting that Ppol is also acting as a barrier against lineal DNA of identical methylation pattern and G + C content than the host.

**Table 1:** Strains used in this work.

Strain	Description	Phenotype/use	Source
*E.coli* DH5α	*supE44* Δ*lacU169 (Φ80 lacZ*Δ*M15) hsdR17*, *recA1*, *endA1*, *gyrA96*, *thi‐1 relA1*	Cloning purposes	Hanahan (1983)
*Tth* HB27	*ATCC BAA‐163/DSM7039*	Wild type	Donated by Prof Y. Koyama
*Tth* HB27*gdh*	HB27 ∆*TTC1211*::*kat*	Kn^R^. Chromosome labelled	Cava *et al*. (2004)
*Tth* HB27*ppolK*	Δ*ppol::kat*	Kn^R^. Hypercompetent	This work
*Tth* HB27*helH*	Δ*hel::hph17*	Hyg^R^. Hypercompetent	This work
*Tth* HB27*ppol*	Δ*ppol*	Hypercompetent	This work
*Tth* HB27ago‐*ppol*	Δ*ago*, Δ*ppol*	Hypercompetent	This work
*Tth* HB27ago	Δ*ago*	Hypercompent	Blesa *et al*. (2015)
*Tth* HB27*pyrE*	Δ*pyrE::kat*	Kn^R^. Chromosome labelled	This work
*Tth HB27ppol‐pyrE*	Δ*ppol*, Δ*pyrE::kat*	hypercompetent, Kn^R^. Chromosome labelled	This work
*Tth HB27‐313*	Δ*TTC0313::hph17*	Hyg^R^. Chromosome labelled	This work
*Tth HB27ppol‐313*	Δ*ppol*, Δ*TTC0313::hph17*	Hypercompetent, Hyg^R^. Chromosome labelled	This work

### Transjugation is not affected by the absence of Ppol

The data above clearly show that Ppol somehow functions as barrier against DNA acquired by natural competence in a way similar to that described for ThAgo. As ThAgo interference is not active when the DNA is transferred from another *Thermus* strain by transjugation (Blesa *et al*., 2015), we carried out transjugation experiments between mating partners defective in the Ppol protein, as well as with parental strains expressing the Ppol protein. As shown in Fig. 6 (“transjugation“), the transfer of the Kn^R^ marker between these Ppol‐defective strains was similar to that detected between Ppol‐positive partners. As expected from the bidirectional character of the transjugation, where both mates can act as donors and recipients (Blesa *et al*. 2015), the results of mating experiments in which only one of the partners contained the Δ*ppol* mutation were similar (Supporting Information Fig. S6) to the transjugation result in Fig. 6.

In conclusion, the activity of Ppol as barrier shown in Fig. 5 is by‐passed when the DNA is directly donated from another *T. thermophilus* by transjugation, also a similar behaviour to that of mutants lacking the ThAgo protein.

## Discussion

Bifunctional primase‐polymerases of prokaryotes are generally associated to MGE, where they have been proposed to be involved in their replication, concomitant or prior to their horizontal transfer (Kazlauskas *et al*., 2018). However, these unique enzymes could also provide their prokaryotic hosts with reinforced capability to withstand DNA replication damages by re‐starting DNA synthesis on ssDNA segments after, for example, DNA fork stalling, as shown for enzymes of this family from eukaryotes (Guilliam *et al*., 2015). In this work, we have analysed the function of a thermostable Ppol encoded by the mobile genetic element ICETh2 in the extreme thermophile *T. thermophilus*.

As a first step in this analysis, we identified among the *Thermales* group with full genomes assembled, a collection of putative mobile elements that shared with ICETh2 the presence of homologues of Ppol, the protein Hel encoded downstream, that shows similarities to the helicase domain of the DnaG primase, and the Int2 tyrosine recombinase, relevant for the excision and the insertion of these elements. Interestingly, among the putatively ICEs identified in the genus *Thermus*, the Ppol and the Hel proteins appeared as separate genes, whereas in those identified in the genus *Meiothermus* a single protein was found containing a N‐terminal Primpol domain and a C‐terminal DUF927 domain, shown to have helicase activity in a homologue from *Staphylococcus aureus* (Mir‐Sanchis *et al*., 2016). This combination of Primase and Helicase activities in a single polypeptide are typical traits of replicative enzymes from the archaeal primase polymerase group (AEP) (Guilliam *et al*., 2015) and suggested a replicative role for Ppol in ICETh2, likely after its excision from the chromosome.

Contrary to this expectation, an insertion mutant lacking the *ppol* gene did not show any decrease in copy number of the excised form of ICETh2. Moreover, it actually increased the excision of the element respect to the wild type and also increased by twofold to threefold the amount of the circular form over that of the scar left in the chromosome after excision, concomitantly suggesting a Ppol‐independent replication of ICETh2 in the mutant. A much lower increase (twofold) in Circ forms in the *ppol::kat* mutant was also observed in a Δ*ppol* marker‐less deletion mutant (Fig. 4), supporting that most of the increase of the replicative form of ICETh2 detected in the *ppol::kat* mutant was the consequence of polar effects by overexpression of downstream *hel* gene. This was in agreement with the behaviour of a null insertion mutant in the gene immediately downstream (*hel)*, where the increase in the copy number of ICETh2 respect to the scar in the chromosome (Fig. 3) after UV treatment was suppressed. In this context, the Hel protein could likely participate in the replication of ICETh2 after its excision, at least under UV‐stress conditions. However, it is also clear that Ppol is not required for such replication, despite its syntenic association with *hel* gene and the conservation of the *ppol‐hel* pair in ICETh2 homologues support Ppol as a likely partner for replication, a putative tore that contrasts with the low processivity of this enzyme (Pitcher *et al*., 2016). If so, another DNA polymerase has to substitute for Ppol in the replication of ICETh2 under our experimental conditions in the Δ*ppol* mutant. In addition, our experiments also revealed that Ppol was not involved in protection against DNA damage in contrast to the protective role shown for its AEP homologues from eukaryotes (Boldinova *et al*., 2017).

In an extensive search for a physiological role of Ppol, we found that Ppol acted as a barrier against the DNA acquired by the highly efficient natural competence apparatus of this bacterium (Blesa *et al*., 2018), in such a way that mutants lacking Ppol (either insertional or marker free) showed an increased transformability of orders of magnitude, not only with replicative and suicide plasmids (Fig. 5) but also with lineal isogenic DNA (Fig. 6). This apparent protective role of Ppol could be a recruited secondary function selected to protect ICETh2 (where *ppol* gene resides) and its host from new MGEs that could invade the same host. In this sense, in a recent impressive functional screening of metagenomic libraries up to 10 new mechanisms of defence were found (Doron *et al*., 2018), many of which included putative DNA‐interacting proteins (nucleases, putative helicases and others). This suggests that systems annotated as putative replication modules of MGE, like the Ppol‐Hel cluster of ICETh2, would actually be involved in host protection, as already shown in this article for the Ppol protein of *T. thermophilus*.

The way by which Ppol protects the cell against invading DNA is far from being understood. A putative explanation could be its recruitment in the context of the mechanism of DNA–DNA interference defence system provided by ThAgo against low G + C plasmids (Swarts *et al*., 2014) and isogenic lineal DNA (Blesa *et al*., 2015). ThAgo can use a ssDNA guide to search for complementary sequences in eDNA that enters the cell by natural competence, allowing to cut them at positions 10/11 complementary to the 5′ extreme of the guide (Swarts *et al*., 2014). In this activity of ThAgo, the origin of the ssDNA guides remains controversial. It has been shown *in vitro* that ThAgo has a somehow unspecific nuclease activity on dsDNA, baptized as DNA ‘chopping’, which could provide the protein with ssDNA guides after elimination of the complementary DNA strand (Swarts *et al*., 2017). However, this ‘chopping’ model has difficulties to explain the auto‐immunity of the genome, or the way to generate guides on ssDNA, the likely nature of the substrates that enter the cell by natural competence. In this scenario, the ability of Ppol to synthesize small pieces of complementary ssDNA on the incoming ssDNA could provide appropriate guides for ThAgo, and constitute the basis for the apparent similarities between the high‐transformation phenotypes of Δ*ppol* and Δ*ago* mutants. Also, the apparent synergic effects of absence of both proteins in the copy number of the replicative form of ICETh2 (Fig. 4) could be explained through a putative collaboration between both proteins in limiting the replication of plasmids, so far described only for ThAgo (Swarts *et al*., 2014). In this scenario, the moderate increase in the Circ form of ICETh2 in the Δ*ppol* mutant (twofold) could be consequence of an increase in the expression of Hel (i.e. smaller mRNA from a common promoter), and the synergistic effect with ThAgo could be the consequence of a more efficient guide generation during replication of ICETh2 mediated by Ppol. Alternatively, Ppol could participate in some class of control through an independent pathway putatively related to a defensive role of this protein (see below).

The absence of protective effect of single *ppol* and *ago* mutations when the DNA is transferred from other *Thermus* strains by transjugation could also be the consequence of a hypothetical different nature of the transferred DNA as dsDNA. In this context, it is relevant to note that unconventional conjugative processes like that of *Streptomyces* spp could transfer dsDNA instead of ssDNA. Actually, the conjugation apparatus in this genus consists of a single DNA translocase (TraB) that can accommodate and transfer dsDNA to the recipient cell (Thoma and Muth, 2015), and that a similar translocase (TdtA) encoded by ICETh1 is responsible for the transjugation process in *T. thermophilus* HB27 (Blesa *et al*., 2017). However, alternative possibilities exist regarding putative common mechanisms of regulation of the activities of Ppol and ThAgo that could allow the discrimination between eDNA acquired by transformation and DNA transferred by transjugation.

In any case, it is relevant to note that this putative activity of Ppol as guide‐provider needed for ThAgo could be a recruited property, and not the product of coevolved genes, since Ppol distribution does not fully match that of ThAgo proteins across the few sequenced *Thermus* spp genomes (Supporting Information Table S1), being more common the presence of TthAgo (specifically in megaplasmids) than that of Ppol. Actually, a more likely mechanism than DNA chopping for the generation of ssDNA guides in collaboration with recombination proteins has been shown recently for the *Clostridium butyricum* Ago protein in *E. coli* (Kuzmenko *et al*., 2020). Also, the role of ThAgo in the physiology of *Thermus* seems to be much more relevant than DNA invasion defence as it has been involved in chromosomal segregation (Jolly *et al*., 2019), and Δ*ago* mutants show a somehow stressed‐like phenotype differences respect to the wild type like an increased number of pili an motility (Blesa, 2016). In this sense, the presence of Ppol encoded by ICETh2 within a ThAgo containing genetic context could mean a more intense protection against certain types of DNA, as observed with the transformability of integrative plasmid (Fig. 5).

In addition to a putative collaboration between Ppol and ThAgo, our experiments also point to a defensive role for Ppol independent of ThAgo, since the strain lacking Ppol produced clearly more transformants than the Δ*ago* mutant when suicide plasmids were used (Fig. 5). A putative explanation for this could be based on the ability of Ppol to compete with recombination proteins (DprA and RecA) for ssDNA binding and convert the ssDNA required for recombination to dsDNA, thus leading to interference in the recombination process itself.

Whatever the way in which the presence of Ppol interferes with eDNA, either in collaboration with ThAgo, by interference with recombination enzymes, or by other yet non‐predicted means, the data presented in this work clearly support that Ppol is involved in defence against invading eDNA, likely protecting the host from new MGE.

## Experimental procedures

### Strains and growth conditions

The strains used and isolated along this work are described in Table 1. Escherichia *coli* was grown at 37°C under stirring in liquid Luria Bertani (LB) medium or in the corresponding agar plates. *Thermus thermophilus* was grown at 60 or 65°C in TB (Trypticase 8 g/l, Yeast extract 4 g/l, NaCl 3 g/l in carbonate‐rich mineral water) under rotational shaking (180 rpm) or in 2% (w/v) agar plates. Kanamycin (Kn, 30 μg ml^−1^), Ampicillin (Amp, 100 μg ml^−1^), or Hygromycin B (Hyg, 100 μg ml^−1^) were used for selection.

### Isolation of mutants

Al the cloning and gene constructions were first amplified in *Escherichia coli* DH5α and then transferred to *Thermus thermophilus*. The plasmids used in this work are described in Table 2 Oligonucleotides used as primers in PCR and qPCR are described in the Supporting Information Table S2.

Insertion knockout mutants were selected by placing either the *kat* or the *hyg* gene cassette encoding Kn^R^ or Hyg^R^ between upstream and downstream 0.9–1 kbp‐long recombination arms respect to the targeted gene. The constructed plasmids were transformed in the desired strain of *T. thermophilus*, and antibiotic resistant mutants were selected on TB plates (Table 2). Resistant clones were streaked twice on selection plates to avoid the presence of wild‐type copies of the targeted gene in this polyploid bacterium (Ohtani *et al*., 2010) to finally obtain *∆gene::kat* or *∆gene::hyg* mutant.

**Table 2:** Plasmids used in this work.

Plasmid	Description/use	References
pMotH	Bifunctional ^a^ modular vecto*r*. Hyg^R^	Verdu *et al*. (2019)
pMotK	Bifunctional^a^ modular vector. Kn^R^	Verdu *et al*. (2019)
pyrEK	Suicide plasmid in *Tth*. Amp^R^ (*Eco*), Kn^R^	This work
pUC19	Cloning in *Eco* of constructs for insertion mutants. Amp^R^	Vieira and Messing (1982)
p313H	pUC19::*TTC0313*::*hph17*. Up and down arms for *TTC0313* mutation by insertion of Hyg resistance cassette	This work
pIB070	pUC19::Δ*TTC0657*::*hph17*. Up and down arms for *TTC0657* mutation of helicase by insertion of Hyg resistance cassette	This work
pD2lox	Suicide in *Tth*. Deletion of *ppol*. Amp^R^(*Eco*), Kn^R^	This work
p174Cre	Bifunctional^a^. Expression of CRE in *Tth*. Hyg^R^	This work

^a^ Plasmids that replicate in *E. coli* (*Eco*) and in *T. thermophilus* (*Tth*).

For the selection of clean Δ*ppol* mutants, a Cre‐lox‐based strategy was followed (Togawa *et al*., 2018). In essence, a gene cassette conferring thermostable resistance to kanamycin bordered by *lox* sites 66 and 71 (Lambert *et al*., 2007) was placed between upstream and downstream flanking arms respect to the *ppol* gene to obtain plasmid pD2‐lox, which was then used for selection of Kn‐resistant mutants (Δ*ppol::kat*) by homologous recombination. The Kn^R^ mutants were further transformed at 60°C with plasmid p174Cre which expresses the Cre recombinase as a transcriptional fusion to a Hyg^R^ cassette. Plasmid p174Cre is a derivative of pMH18 in which the gene coding for the Cre recombinase was cloned between its Xba I and EcoR I sites after its amplification with primers NCreXba and CCreXba (Supporting Information Table S3) from plasmid pCI‐cre (a kind gift from Fabio Rossi).Hyg^R^ and Kn^R^ colonies were further streaked on plates with only Hyg. After 5.5 days of growth at 50°C to allow the expression of thermosensitive Cre recombinase individual colonies resistant only to Hyg were selected. The absence of the *ppol* and the *kat* genes in these colonies was confirmed by PCR with the appropriate primers (Supporting Information Table S3) and then they were further grown at 65°C in liquid TB without antibiotics to allow the spontaneous loss of the p174Cre plasmid. Individual clones sensitive to both Kn and Hyg were selected and checked for the absence of the p174CRE plasmid and the *ppol* gene by PCR and sequencing. Clones Δ*ppol*‐5 and Δ*ago*‐Δ*ppol*‐2 were selected for furthers studies.

### Transformation and transjugation assays

The desired amount (20 or 100 ng) of DNA was added to 0.5 ml of mid‐exponential cultures of *T. thermophilus*. After 4 h incubation at 60°C the cells were spread on selection plates, which were then incubated at the required temperature. Transformation frequencies were calculated as the number of CFU on selective plates per viable cells. For transjugation assays, 100 μl of overnight cultures of the Hyg^R^ or Kn^R^ parental strains were mixed in presence DNaseI (5 units; Roche), washed by mild centrifugation at room temperature with the same volume of TB, and re‐suspended in 10 μl of TB with Dnase I (5 units; Roche). The cell mix was applied onto nitrocellulose filters (GSWP, Millipore) on TB plates. After 4 h of incubation at 60°C, cells were extracted by vigorous shaking in 1 ml of TB and appropriate dilutions were plated on pre‐warm selective plates containing the required selection antibiotics. Transjugation frequencies were expressed as CFU of double resistant colonies per CFU of each parental (Kn^R^ or Hyg^R^).

### UV treatment and qPCR

Cultures at stationary phase were diluted to OD600 ≈0.05 and grown on TB. When cultures reached OD600 ≈0.4, 2 ml of each culture were exposed to ultraviolet radiation (60 J m^−2^) using an UV lamp (Sylvania OSRAM StIII, Germany) for 20 min. Then both UV exposed and control cultures were incubated at 65°C under shaking for 3 h in the absence of light. Cells were pelleted and total DNA extracted as follows.

Genomic DNA was extracted using DNeasy® blood and tissue kit (Qiagen). qPCR assays were carried out in 384‐well plates in a final volume of 10 μl. Reference genes used were the 16S rRNA (*TTC3084*), the DNA polymerase III (*TTC1806*) and the RNA polymerase alpha subunit (*TTC1300*). Primers used for amplification are indicated in the Supporting Information Table S3. The mastermix reagents used were: 1 μl of primer mix (5 μM of each primer) + 5 μl *Power* SYBR® Green PCR Master Mix (Thermo Fisher Scientific, CN 4367659) which includes AmpliTaq Gold® DNA Polymerase DNA polymerase, dNTPs and the rest of components needed to perform the PCR. The equipment used was a CFX384 Real Time System C1000 Thermal Cycler (Bio‐Rad), in hard‐Shell® 384‐Well PCR Plates White Well Clear shell (Bio‐Rad CN HSP‐3805). Technical triplicates of each sample were performed and at least four experimental replicas. The efficiency value used was the obtained in the standard curves.

To study the effects of UV on the survival of the cells, cultures at stationary phase grown at 60 or 65°C were diluted to OD_600_ ≈0.05 and grown on TB up to OD_600_ ≈0.3 at 60 or 65°C. Then 8 μl of serial dilutions were drop‐inoculated on plates that were further irradiated with UV (60 J m^−2^) using an UV lamp (Sylvania OSRAM StIII, Germany) for 5, 10 and 15 s, keeping also untreated control plates. The plates were further incubated in the dark at 60°C or 65°C for 72 and 48 h, respectively, to allow the growth of the surviving cells.

PCR relative quantification of the data was carried out using software GenEx v.5.4.4. (MultiD Analyses AB, Gothenburg, Sweden). Data absolute quantification processing was carried out using software Microsoft Excell 2010 (after correction of efficiency of Cq values in software Genex).

## Supporting information


**Appendix S1** Supporting Information.
