# Supplementary material for: A thermostable DNA primase‐polymerase from a mobile genetic element involved in defence against environmental DNA
Source: Environ Microbiol. 2020 Sep 3;22(11):4647–57. doi: 10.1111/1462-2920.15207 (PMC7693054; doi:10.1111/1462-2920.15207)
Supplement: Supplementary file 1 — Appendix S1 Supporting Information. [file EMI-22-4647-s001.docx]

**Supplementary material to:**

**A thermostable DNA primase-polymerase from a mobile genetic element involved in defence against environmental DNA**

Nieves García-Quintáns, Ignacio Baquedano, Alba Blesa^2^, Carlos Verdú, José Berenguer* and Mario Mencía*

^1^ Centro de Biología Molecular Severo Ochoa (CBMSO), Universidad Autónoma de Madrid-Consejo Superior de Investigaciones Científicas, Madrid 28049, Spain

^2^ Department of Biotechnology, Faculty of Experimental Sciences, Universidad Francisco de Vitoria, Madrid, 28223, Spain

***** Correspondence: jberenguer@cbm.csic.es; [mmencia@cbm.csic.es](mailto:mmencia@cbm.csic.es); Tel.: +34-911-964-498

Short title: A primase polymerase protects the host from eDNA.

**Supplementary Table 1.** Co-occurrence of genes used for the ICE-like architecture search (Ppol, Hel and Int2 homologues) and genes coding for the Argonaute ThAgo homologues, found among Thermales and other thermophiles. Search was done using PSIBLAST and tBLAStn within the NCBI database using *T.thermophilus* HB27 genes (locus indicated in the first row) as template.

| **Strain** | **Ppol (TTC0656)** | **Helicase (TTC0657)** | **Integrase (TTC0665)** | **Argonaute (TTP0026)** |
| --- | --- | --- | --- | --- |
| *Thermus thermophilus HB27* | x | x | x | x |
| *Thermus thermophilus TMY* | x | x* | x | x |
| *Thermus thermophilus SG0.5 JP16-17* | x | x | x | x |
| *Thermus thermophilus HC11* | xx | x* | x | x |
| *Thermus thermophilus AA2-20* | x | x* | x | x |
| *Thermus thermophilus AA2-29* | x | x* | x | x |
| *Thermus thermophilus HB8* |  | x | x | x |
| *Thermus thermophilus JL18* |  |  | x | x |
| *Thermus thermophilus NAR* |  |  | x | x |
| *Thermus aquaticus Y51MC23* | x | x | x | x |
| *Thermus oshimai JL2* | x | x | x |  |
| *Thermus scotoductus SA01* | xx |  | x | x |
| *Thermus parvatiensis* |  |  | x | x |
| *Thermus brockianus* |  |  | x | x |
| *Thermus CCB_US3_UF1* |  |  | x | x |
| *Thermus amyloliquefaciens* | x |  |  |  |
| *Meiothermus silvanus DSM9946* | x |  | x |  |
| *Meiothermus ruber DSM1279* | x |  | x |  |
| *Meiothermus ruber DSM1280* | x |  | x |  |
| *Meiothermus taiwanensis WR-220* | x |  | x |  |
| *Rubrobacter xylanophilus DSM 9941* |  | xx | x | x** |
| *Rubrobacter xylanophilus AA3-22* |  | x | x |  |
| *Rubrobacter indicoceani SCSIO 08198* |  | x | x |  |
| *Moorella thermoacetica strain DSM 521* |  | x | x |  |
| *Moorella thermoacetica strain DSM 2955* |  | x | x |  |
| *Moorella thermoacetica ATCC 39073* |  | x | x |  |
| *Moorella thermoacetica 39073 HH* |  | x | x |  |

*) A putative helicase with low sequence similarity to TTC0657 is encoded after the Ppol homologue; * *) identity < 30%

**Supplementary Table 2**. Oligonucleotides used in this work

| **Name** | **Use** | **Sequence (5’->3’)** |
| --- | --- | --- |
| 195-Fw | PCR Up-primpol | AAAGAATTCCTCTGGAAAGCTGGGCTCG |
| 268-Rv | PCR Down-primpol and sequencing | AAAGAATTCCTTGAGGAGGTCTTGGAAGG |
| 267-Rv | PCR Down-primpol | AAAGAATTCCTTCCGCTTGAGGGCCAG |
| 184-Fw | PCR into primpol | CCACCCTCGAGGCCTGGTG |
| 183-Rv | PCR into primpol | GACCTCCTCCGCTTCCCTG |
| Kat1 | PCR test cassette insertion | CCTTTTTCCCCGCATCC |
| Kat-3 | PCR test *kat* in Δppol | GGAACGAATATTGGATA |
| Kat-4 | PCR test *kat* in Δppol | AGAAATTCTCTAGCGAT |
| Hy-seq-Fw | PCR test *hph17* | CGATTGCTGATCCCCATGTG |
| Hy-cre-Rv | PCR test *hph17* | CAGTGCGTTCGAACGCTAGAG |
| Check_TTC0313_Fw | PCR test cassette insertion in *TTC0313* | GGCGCCTGCCCCTACC |
| ChrPyrEFw | PCR test cassette insertion in *pyrE* | GTGACTGGGGCGAAGTCGTAAC |
| PPol3 Fw | qPCR putative reference gene | CCGAACGCCTTCTTCTACTC |
| PPol3 Rv | qPCR putative reference gene | GTGGACGTAGGCTTTGACCT |
| PRNAPol Fw | qPCR putative reference gene | GCAGCTGGTGATCGAGTTCT |
| PRNAPol Rv | qPCR putative reference gene | GGGCCTTGAGCTTGGAAT |
| P16S Fw | qPCR putative reference gene | CTCGCAAGCCTTGACAAAAAG |
| P16S Rv | qPCR putative reference gene | GCAGCAAAAGCCATGCTATCA |
| P435 | Detection of ICETh2 *attB2* by qPCR | CGAGAAGGAGGTGTGGAAC |
| P438 | Detection of ICETh2 *attB2* by qPCR | TGCCCGGTGTCGTAGA |
| P453 | Detection of ICETh2 *attL2* by qPCR | TCGCCGCAATGGAGTTGT |
| P454 | Detection of ICETh2 *attL2* by qPCR | CCCGAAAATGACCGGCT |
| P488 | Detection of ICETh2 *attI2* by qPCR | AAGCTTTGTGTTCTGATGCTGG |
| P489 | Detection of ICETh2 *attI*2 by qPCR | ACGGGAAGAAAAGGGCTAGTG |
| P501 | Construction of pIB070 | AACTGCAGTCATCATAACGCC |
| P502 | Construction of pIB070 | AATCTAGAGAGGTCTTGGAAGG |
| P503 | Construction of pIB070 | AATCTAGAGCCTCTTCAAGGTA |
| P504 | Construction of pIB070 | AAGAATTCGGGCAGACGAACT |
| BglII_hph17_Fw | Construction of pUC19::*TTC0313*::*hph17* | AAAAAGATCTGAAACCTTAAGgcccgacc |
| BglII_hph17_Rv | Construction of pUC19::TTC0313::*hph17* | TTTTAGATCTCTATTCCTTTGCCCTCGG |
| NCreXba | Construction of pMH174Cre | AAATCTAGAGAAGGAGGTCGACATGGCCAATTTACTG |
| CCreXba | Construction of pMH174Cre | TTTGAATTCTTTCAGACAGATCTAAGGCCGCTATCG |


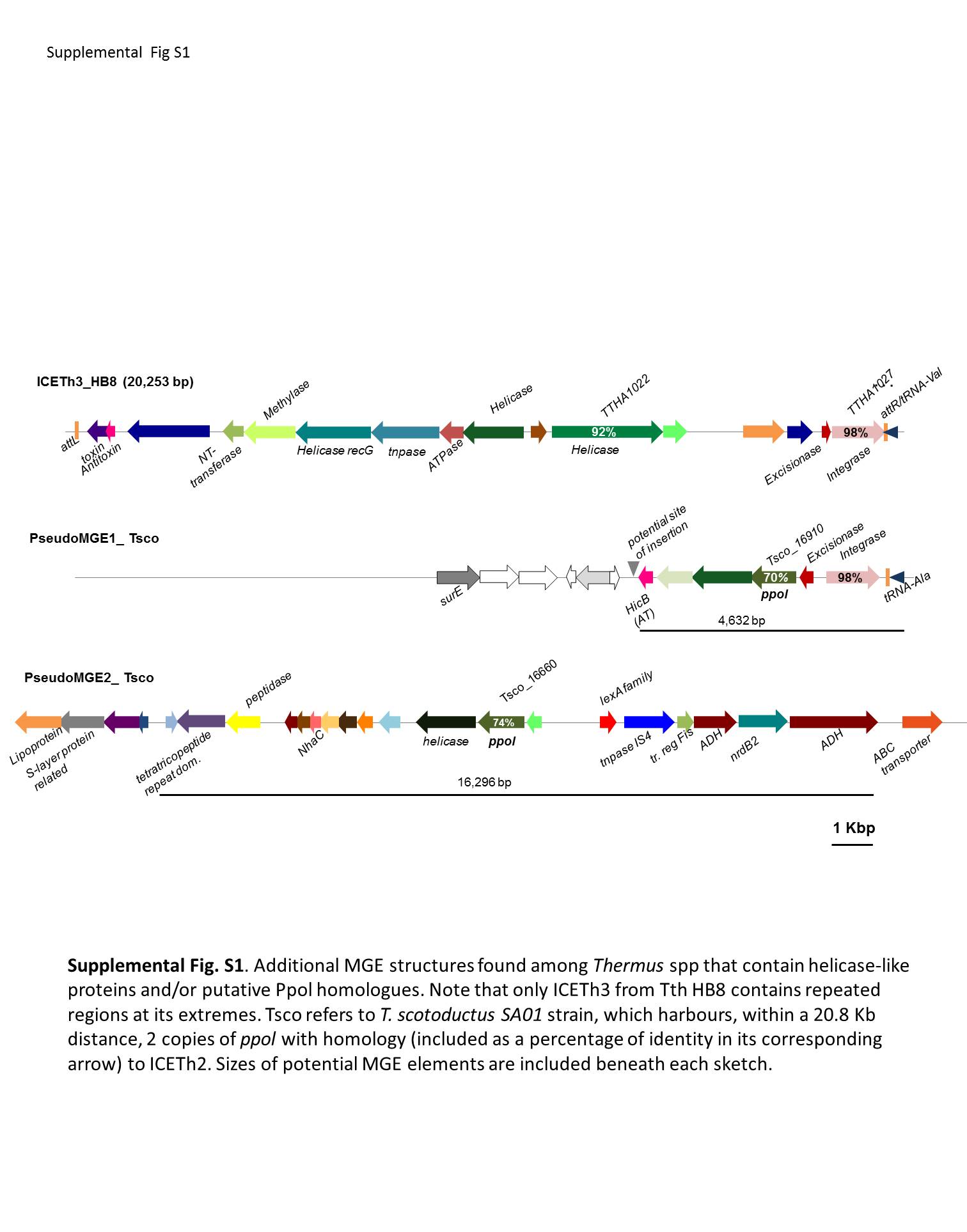


**Supplementary Fig. S1**. Additional MGE structures found among *Thermus* spp that contain helicase-like proteins and/or putative Ppol homologues. Note that only ICETh3 from Tth HB8 contains repeated regions at its extremes. Tsco refers to *T. scotoductus SA01* strain, which harbours, within a 20.8 Kb distance, 2 copies of *ppol* with sequence homology, included as a percentage of identity in its corresponding arrow, to ICETh2 *ppol*. Sizes of potential MGE elements have been estimated and are included beneath each sketch.


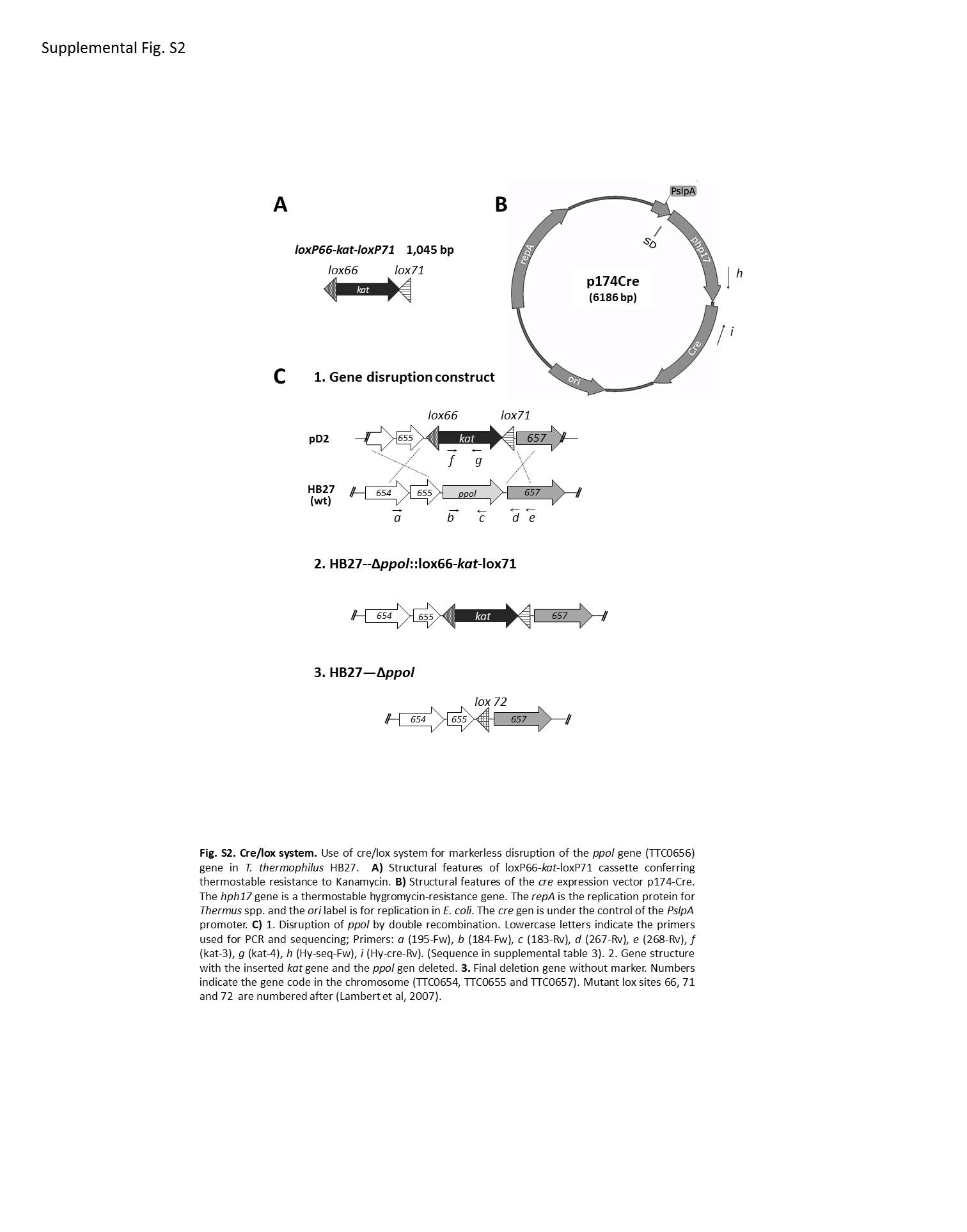


**Supplementary Fig. S2.** Use of Cre/*lox* system for markerless disruption of the *ppol* gene (TTC0656) gene in *T. thermophilus* HB27. **A)** Structural features of loxP66-*kat*-loxP71 cassette conferring Kn^R^. **B)** Structural features of the *cre* expression vector p174-Cre. The *hph17* confers thermostable Hyg^R^. The *repA* codes for the replication initiation protein for the plasmid in *Thermus* spp, and the *ori* labels the replicative origin for *E. coli*. The recombinase *cre* gen is under the control of the *PslpA* promoter. **C) 1.** Disruption of *ppol* gene through homologous recombination. Lowercase letters indicate the primers used for PCR and sequencing; Primers: *a* (195-Fw), *b* (184-Fw), *c* (183-Rv), *d* (267-Rv), *e* (268-Rv), *f* (kat-3), *g* (kat-4), *h* (Hy-seq-Fw), *i* (Hy-cre-Rv) (sequences in Supplemental Table 3). **2.** Gene structure with the inserted *kat* gene replacing the *ppol* gene. **3.** Final structure of the genome region of the Δ*ppol* mutant. Numbers inside the arrows indicate the gene codes in Tth HB27 genome (TTC0654, TTC0655 and TTC0657). Mutant *lox* sites 66, 71 and 72 are numbered after (Lambert et al., 2007).





**Supplemental Fig. S3. Growth rate of the *Δppol* mutant.** The wild type and *Δppol* strains were grown for 24 hours at 60, 65 and 70°C. The duplication time (Dt) time is calculated in the exponential phase of growth.


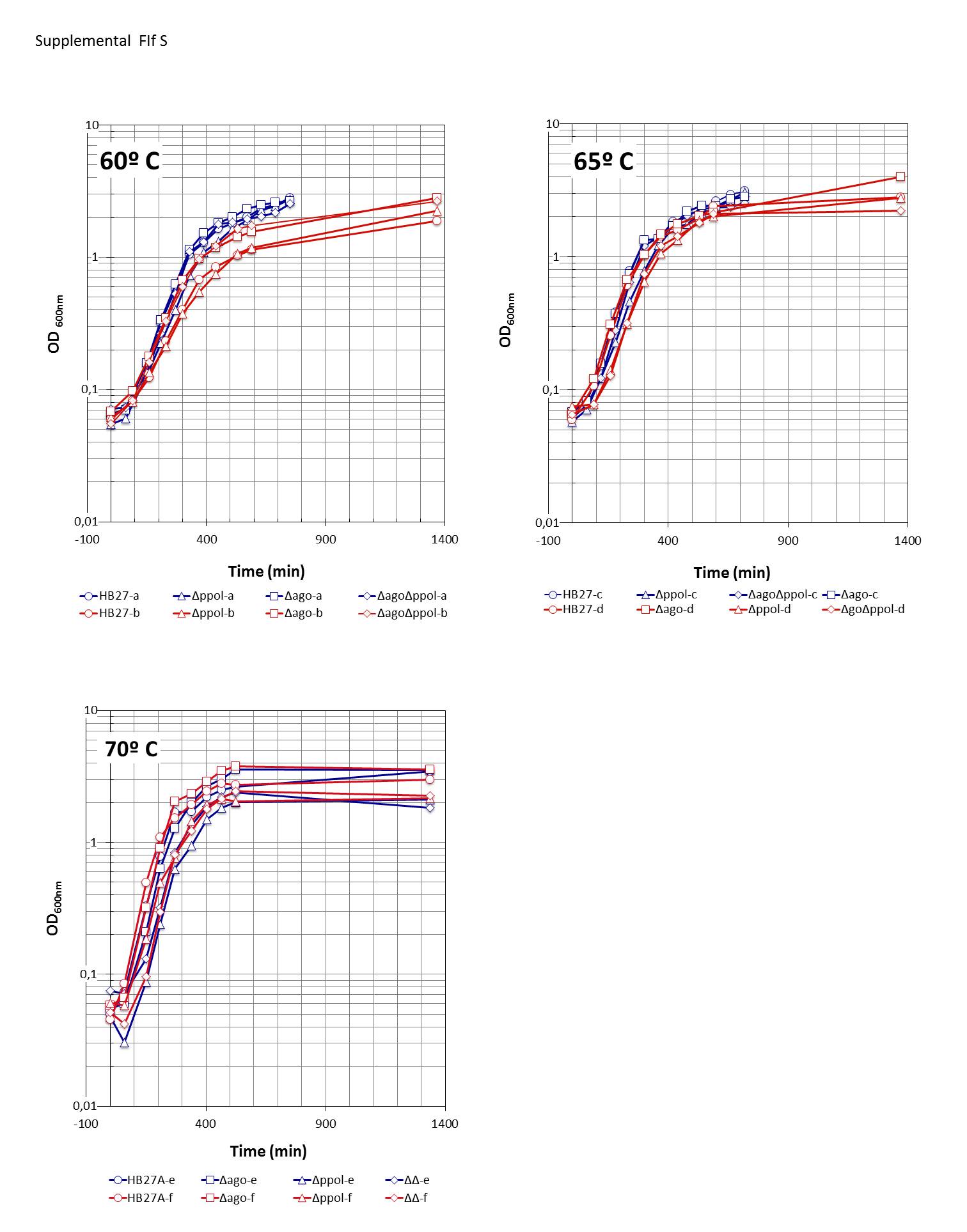


**Supplementary Fig. S4.** Growth of will type and mutants strains at different temperatures. Panels show the growth of the wild type strain HB27 (empty circles) the Δ*ppol* (empty triangles), the Δ*ago* (empty squares) and the double *Δago-Δppol* mutant (empty diamonds) at the indicated temperatures in two independent experiments (red and blue lines).





**Supplemental Fig. S5. Effect of stressors in the Δ*ppol* mutant.** The wild type strain (HB27) and its derivative mutants *Δppol*, *Δago* and *Δppol*-*Δago*, were subjected to different stress conditions that affect DNA. A) Effect of UV radiation. The cell cultures were grown at the indicated temperatures until an OD_600_ of 0.3 was reached (3x10^8^ cell/mL). Then, 8 μl of serial decimal dilutions were drop-inoculated on plates that were further irradiated with UV for 5, 10, and 15 seconds. The plates were incubated in the dark at the indicated temperatures. B) Resistance to DNA damaging agents. Sterile disc papers were soaked with different amounts (in μg) of Mytomycin (Mito), Bleomycin (Bleo), Novobiocin (Novo), Ciprofloxacin (CFX) and hydrogen peroxide (H_2_O_2,_ in % v/v), and placed on TB plates previously spread-inoculated with 10^7^ cell/ml of each of the mutants. The plates were incubated at 60°C for 24 hours in the dark. Inhibition halos were measured and plotted on a radial graph. C) Representative image of the results obtained of assays with 40 μg (disc 1) or, 60 μg (disc 2) of Novobiocin, and 30 μg (disc 3) or 60 μg (disc 4) of Ciprofloxacin.


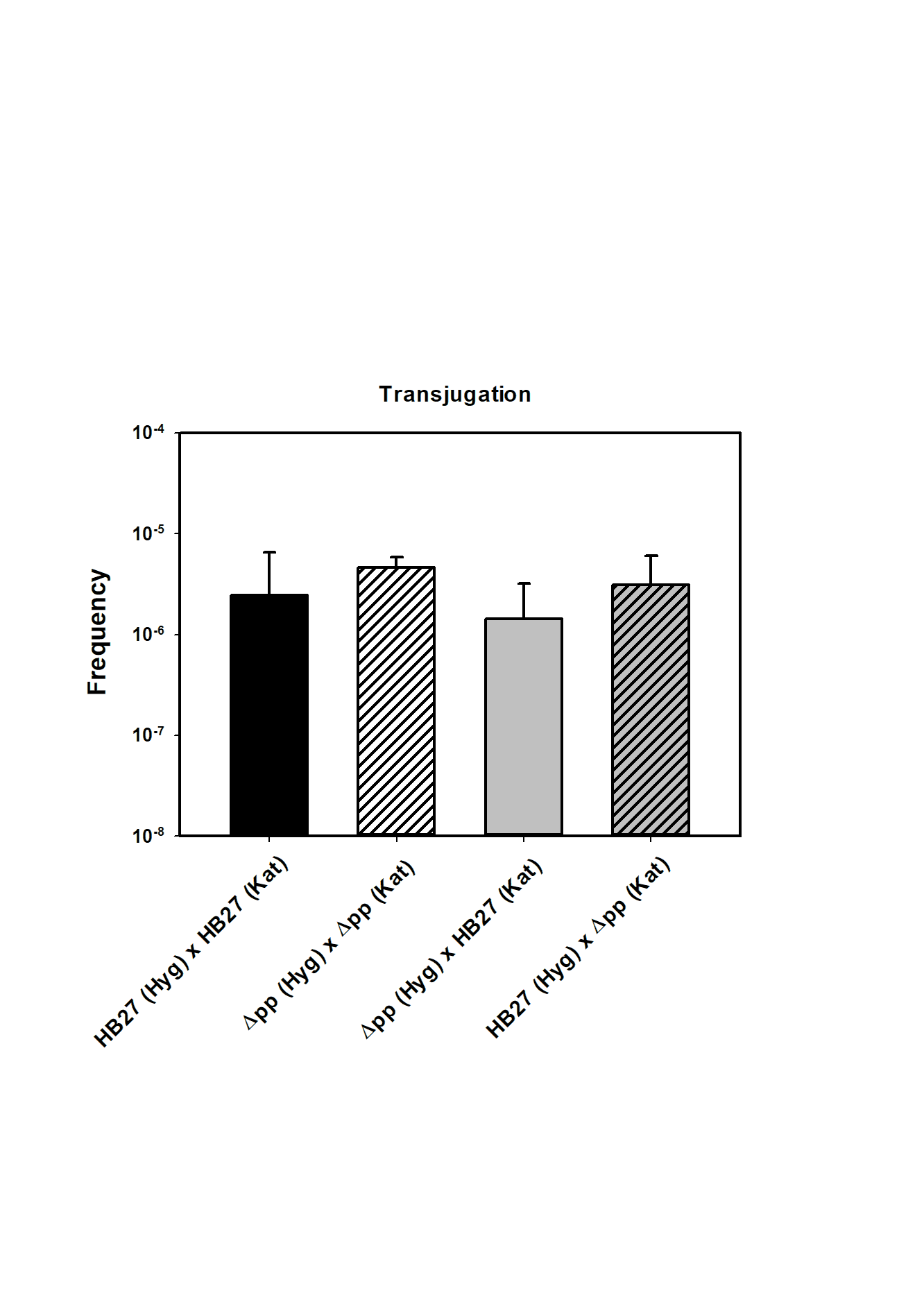


**Supplemental Fig. S6. The absence of Ppol in any of the mates in transjugation experiments does not affect to the DNA transfer.** Two TTH HB27 derivative strains one labelled at the *pyrE* locus with kanamycin resistance (Kat) and the other labelled at locus TTC0313 with hygromycin resistance (Hyg) were used in mating experiments in which none (Black), one (grey), or both (striped white) mates were devoid of the Ppol protein. Transjugation frequencies were calculated as the ratio between the number of double resistant colonies (Kn^R^ plus Hyg^R^) and Kn^R^ colonies. Frequencies are the average of 3 independent experiments, and error bars correspond to standard deviation. The statistical differences between the different combinations were not significant (p-value > 0.5).

**References to supplementary material**

Lambert, J.M., Bongers, R.S., and Kleerebezem, M. (2007) Cre-lox-based system for multiple gene deletions and selectable-marker removal in *Lactobacillus plantarum*. *Appl Environ Microbiol* **73**: 1126-1135.
